# One-Class Convolutional Neural Networks for Water-Level Anomaly Detection

**DOI:** 10.3390/s22228764

**Published:** 2022-11-13

**Authors:** Isack Thomas Nicholaus, Jun-Seoung Lee, Dae-Ki Kang

**Affiliations:** 1 Department of Computer Engineering, Dongseo University, 47 Jurye-ro, Sasang-gu, Busan 47011, Republic of Korea; 2 Infranics R&D Center, 12th flr. KT Mok-Dong Tower 201 Mokdongseo-ro, Yangcheon-gu, Seoul 07994, Republic of Korea

**Keywords:** convolutional neural network, one-class classification, anomaly detection, water-level anomaly, synthetic data

## Abstract

Companies that own water systems to provide water storage and distribution services always strive to enhance and efficiently distribute water to different places for various purposes. However, these water systems are likely to face problems ranging from leakage to destruction of infrastructures, leading to economic and life losses. Thus, apprehending the nature of abnormalities that may interrupt or aggravate the service or cause the destruction is at the core of their business model. Normally, companies use sensor networks to monitor these systems and record operational data including any fluctuations in water levels considered abnormalities. Detecting abnormalities allows water companies to enhance the service’s sustainability, quality, and affordability. This study investigates a 2D-CNN-based method for detecting water-level abnormalities as time-series anomaly pattern detection in the One-Class Classification (OCC) problem. Moreover, since abnormal data are usually scarce or unavailable, we explored a cheap method to generate synthetic temporal data and use them as a target class in addition to the normal data to train the CNN model for feature extraction and classification. These settings allow us to train a model to learn relevant pattern representations of the given classes in a binary classification fashion using cross-entropy loss. The ultimate goal of these investigations is to determine if any 2D-CNN-based model can be trained from scratch or if transfer learning of any pre-trained CNN model can be partially trained and used as the base network for one-class classification. The evaluation of the proposed One-Class CNN and previous approaches have shown that our approach has outperformed several state-of-the-art approaches by a significant margin. Additionally, in this paper, we mention two interesting findings: using synthetic data as the pseudo-class is a promising direction, and transfer learning should be dealt with considering that underfitting can happen because the transferred model is too complicated for training data.

## 1. Introduction

Water systems are essential infrastructures for water quality for the social quality of life and sustainable economic activities. Considering the importance of water systems, water storage and distribution systems play a vital role in human life and economic growth. Therefore, appropriate measures should be taken to protect those water systems from any threat that can damage them and lead to significant economic and environmental issues. That is why understanding the abrupt changes or fluctuations in water levels is necessary to safeguard and sustainably manage water systems effectively.

An essential task in managing these systems is detecting fluctuations or anomalies in particular time steps and reporting them so that the operators can take necessary actions to resolve underlying issues. For example, an anomaly score can be produced based on sensor data and used as an indicator of a power plant failure [1]. Precise detection is crucial to prevent serious business crisis and losses, as it is reported that one minute of downtime of an automotive manufacturing plant may cost thousands of dollars [2]. Much research has been conducted to develop a sophisticated data-driven solution (e.g., machine- and deep-learning-based approaches) because of the availability of such data and the issue’s importance.

Recently, deep-learning-based techniques have dominated traditional techniques for a significant margin in various domains and industrial applications. The deep-learning techniques are the advanced version of biologically (more precisely, of the human brain)-inspired techniques [3] known as neural networks. Since the 2010s, when deep-learning-based techniques became popular [4], most time-series anomaly detections were tackled using traditional techniques such as rule-based techniques (e.g., [5,6,7]) and classical machine learning [8,9,10]. In recent years, advancements in deep learning have revolutionized the scope of data-driven modeling use cases. Two primary network architectures led to this marginal advancement: Convolutional neural networks (CNN) [11] and Recurrent neural networks (RNN) [12]. Initially, RNNs were developed for temporal sequence tasks [13,14,15] and CNNs for image-related tasks [16,17,18].

We recognized the potential of convolutional neural networks as they have proven to produce competitive methods in various tasks, including computer vision tasks [19], natural language processing tasks [20], and even time-series domains [21,22]. The benefit of using CNNs for sequence classification [23] is that they can learn from the raw temporal data directly and do not need domain expertise to extract input features manually. The model generated from CNNs for sequence classification can learn an internal representation of the temporal data and ideally achieve comparable performance to models fit on a version of the dataset with engineered features.

**Figure 1:**
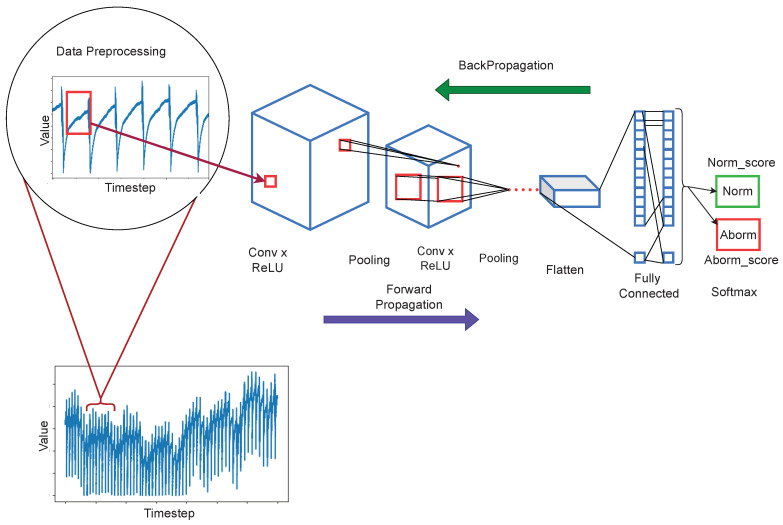
The illustration of steps followed from data to input to the final classification of each given data sequence converted to raw pixel images and fed to the end-to-end anomaly detection system built using a 2D-Convolutional Neural Network architecture that outputs the class of the given input sequence.

However, it has been difficult to apply CNN for detecting anomaly by modeling normal behavior only [24], because CNN is basically a discriminative model. In the real world, it is very difficult to obtain anomalous data because it is from rare events, whereas it is very easy to obtain normal data which are mostly present. Therefore, in our study, we adopt one-class neural network approaches [25,26] to model the anomalous behavior of water level. Considering all the benefits of CNN models, our approach is also built on 2D CNN and trained on normal and synthetic data to learn a decision boundary that will be sufficient to distinguish the two classes during the inference phase. We start by preparing the synthetic data through a guided generative process using a synthetic time-series data generation library. After that, we preprocess both sensors’ (normal) and synthetic data (anomaly) and then use them to fit the model. We used Adam optimizer [27] and binary cross-entropy loss function to train the model to learn a decision boundary from relevant feature representations of the data. We successfully produced a competitive model for anomaly detection following those steps (also illustrated in Figure 1).

The following are the contributions of our study:

- We extensively investigated the potential of 2D Convolutional Neural Networks for temporal data one-class classification. We aimed to use CNN models as feature extractors and classifiers to build an end-to-end anomaly detection system. We successfully trained the system on normal (collected sensor) and abnormal (synthetic generated) data, then evaluated it using test data containing both normal and abnormal data points.
- We explored the knowledge transfer across domains that is between two unrelated tasks. This task intended to explore a similar learning paradigm as that of transfer learning between the image classification and natural language processing. The difference is that we used fully trained models for image classification which have never learned time-series data for classification. The OC-CNN achieved our goal by partially training the pre-trained images classification model using our dataset and performing a classification task.
- And we provide detailed experiments, results, analyses, and discussions that give new insights on how to deal with data absence in time-series problems and present the readers with informative findings.

Here is how we organized the rest of the manuscript: Section 2 provides a literature review of the prior works. Section 3 contains a detailed explanation of the methods under investigation. Section 4 describes the experiment. Section 5 provides the results and discussion of the 2D-CNN-based models, Autoregressive Integrated Moving Average (ARIMA) [28], and Hierarchical Stacking Temporal Convolutional Network (HS-TCN) [29] experiments. Lastly, the discussion and conclusions in Section 6 and Section 7, respectively.

## 2. Related Work

Safeguarding the infrastructure facilities is one of the crucial tasks in daily controlling and monitoring system operations. Considering its importance, recently, much work has been focusing on ensuring that faults are detected early and reported immediately. That will provide the chance to avoid damage to the facilities/infrastructure or at least prevent catastrophic events. Despite the effort, fault or anomaly detection for water-level monitoring systems is still under-explored. Thus, we adopted 2D convolution neural networks to classify data points generated by the system as either anomalous or normal. To the best of our knowledge, no prior work adopts 2D convolution neural networks for water-level anomaly detection.

### 2.1. One-Class Classification

One-Class Classification [30] became popular in anomaly detection because of its ability to learn from only one class of data (normal data) and then apply it to binary problems. Currently, One-Class classification is performed using a few classical machine learning, or neural network-based algorithms. For example, a few OCC classical machine learning methods that are widely studied and used are Naïve Bayes Positive Class (NBPC) [31], One-Class Support Vector Machine (OC-SVM) [32], and Support Vector Data Description (SVDD) [33].

Naïve Bayes Positive Class (NBPC) is an extension of a traditional Naive Bayes classifier that attempted to correct the calculation of an instance’s class probability. The NBPC introduced a threshold *t* that checks if the likelihood of an instance’s attribute value is great or equal to *t*, then it belongs to a given class; otherwise, it considers the instance anomalous.

SVM-based methods introduced the concept of finding a boundary that maximizes the margin between two classes and works well for binary and multi-class classification. One of the obstacles for applying SVM to one-class problems is that the negative class data information is usually unavailable. Scholkopf et al. proposed a one-class SVM (OC-SVM) to deal with the issue. Their approach tackles the absence of abnormal class data by maximizing the boundary with respect to the origin.

The Support Vector Data Description (SVDD) presented by Tax and Duin [33] is another popular technique inspired by the SVM, in which a hypersphere encircles the target class data. Various extensions of OC-SVM have been proposed and proven to be a practical approach for numerous case studies.

Unfortunately, despite their ability to discriminate anomalies from normal data, they are not the appropriate solutions for time-series problems because those algorithms are not inherently designed to incorporate the temporal property of time-series data.

Neural network-based approaches have been widely studied and used since the introduction of Deep Neural Networks (DNN). The DNN showed impressive performance in modeling complex tasks such as recognition, object detection, segmentation, key-point detection, etc. Additionally, recently, the DNN methods have been introduced to the anomaly detection field to cope with the challenges encountered when adopting them in the OCC problems. For example, unsupervised methods such as AutoEncoder (AE) [34,35,36], Deep Belief Network (DBN) [37,38,39], and Generative Adversarial Network (GAN) [40,41,42] provide efficient solutions to solve problems involving the absence of positive (abnormal) class data. Oza and Perera [26] introduced approaches closely related to our work because we all use similar styles to counter the problem of training the DNNs for one-class classification. Oza’s idea is to learn a good representation along with a decision boundary using One-Class CNN via the entropy loss function with the help of a pseudo-abnormal class generated using a zero-centered Gaussian noise in a latent space. Perera’s [43] work relies on unrelated task labeled data as an abnormal class to train a CNN model for feature learning in OCC. Compared with the two approaches, we used CNN to learn from temporal collected (normal) and synthetic (abnormal) data.

Time-series anomaly detection is certainly an interesting topic with numerous works in the machine learning research community. Zhu et al. [44] introduce a combination of Long Short-Term Memory (LSTM) and GAN to harness each technique’s significant benefits, leading to excellent performance. Those benefits are the capabilities of LSTM over the temporal data and GAN for data feature extraction and data (normal) model construction. The GAN constructs a normal data model that outputs the generator residual and discriminator loss. Then a threshold is applied to the discriminator loss to judge the given input as anomalous or normal during a test phase. Chadha et al. [45] employ deep autoencoder and Top-K clustering objectives for grouping the latent space based on the most discriminative latent space features.

### 2.2. Transfer Learning

Transfer learning is one of the methods employed in deep learning to accelerate learning by using the knowledge learned from other related tasks to the targeted task to be solved [46]. This learning method became prominent and proved its potential in other domains, mostly in computer vision tasks such as object localization and image recognition, and recently slightly in time-series problems. The challenges of applying such methods in temporal data are the lack of a universal or standard and large-scale dataset [47]. Wen and Keyes [47] demonstrated that transfer learning for time-series anomaly detection could successfully improve a CNN-based model pre-trained on a univariate time-series dataset. However, their approach required large-scale time-series data to train the network from scratch before fine-tuning it on a target dataset.

In our study, we also intended to explore the ability of the deep-learning model to borrow prior knowledge from the different domains to solve a task at hand. More specifically, we wanted to fine-tune the computer vision network’s weights to work with time-series problems. We adopted this approach because we targeted classifying given patterns in raw pixel input as either normal or anomalous rather than forecasting. We employed CNN because integrating it with transfer learning led to many successful applications in two-dimensional image recognition tasks. Finally, sharing weights from different tasks in transfer learning could be more promising than using arbitrary initial weights in neural networks.

## 3. Methodology

In this section, we formally describe the proposed water-level anomaly detection approach. First, we introduce the general Convolution Neural Networks and their primary operations, and then we clarify how we adopted them for time-series One-class classification problems. Figure 1 illustrates the workflow of the proposed 2D-CNN-based approach.

We adopted a 2D Convolutional Neural Network (CNN) as a primary feature learner for this classification problem. As can be seen in Figure 1, the CNNs are composed of at least four (4) layers named input, convolutional (kernel), pooling, and fully connected (classification layer). The input layer receives a raw image (can be a 1-channel (grayscale) or colored (3-channels)) and passes it to the convolutional layer. The convolutional layer consists of a set of kernels (also known as filters) that output a feature map when applied to an input image. Stacking multiple convolutional layers of the models allows them to learn complex features from the images. The pooling layer downsizes the input dimensions to perform abstraction, decreases the number of parameters and relaxes memory requirements, and hence makes it easier to process, which leads to faster training. Two widely used pooling methods are max pooling, and average pooling. Max pooling returns the maximum value from the area covered by the kernel, while average pooling returns the average value from the area covered by the kernel. The fully connected layer is the dense layer consisting of mesh-connected network neurons. This layer uses a traditional Feed-Forward Network architecture, which accepts a one-dimensional input vector of the flattened feature maps and outputs the predictions.

The training mechanism is composed of two phases, the first is forward propagation, and the next is error back-propagation. The forward propagation is the inference mechanism by using the network input, and weights. Then, the CNN uses error back-propagation [48] as an error reduction mechanism to optimize the network weights. The learning principle, used in error back-propagation, is to minimize the errors (such as a sum of the squared errors) by iteratively adjusting the network’s weights and biases based on the errors returned from the output layer back to the input layer.

### 3.1. Synthetic Data Generation

We considered using the TimeSynth library [49], which provides several options to generate different forms of time-series data. These options include several signals and noise types separately defined in three different groups of classes named ‘general’, ‘noise generation’, and ‘signal generation’. Among the options, we explored several relevant classes to generate suitable synthetic signals that closely resemble our sensors’ collected data. Synthesis data generation in time-series is a highly complicated process involving several hyperparameters that must be carefully tuned. Therefore, we use ‘AutoRegressive’ class for signal generation, ‘TimeSampler’ for noise or signal sampling instructions (when and how), and ‘TimeSeries’ classes for handling time-series data sampling.

### 3.2. Anomaly Detector

In this paper, we used two forms of learning: transfer learning with partial training and custom model training from scratch. Since they are both end-to-end systems, we feed in the raw input and obtain the prediction to which the instance belongs. The systems operate over the probability distribution of the classes space with SoftMax function. The highest probability from the distribution indicates that the systems regard the associated class of the given input.

#### 3.2.1. Transfer Learning

We chose to use pre-trained ResNet and then replaced the fully connected layer with a layer suitable for binary classification problem. We consider effectiveness and efficiency as the main criteria for adopting this framework. However, one important problem of applying transfer learning of pre-trained ResNet to our anomaly detection task is that the selected pre-trained model has never seen temporal data. Therefore, we decided to partially train the model with our data for a few epochs. That would allow the model to capture some of the informative features from the target temporal data, and then, solve our task.

#### 3.2.2. Custom Model

We designed our model that contained a maximum of four convectional layers with several dropout layers as a regularizer. We kept it simple to avoid underfitting and overfitting of the model. We trained the model from scratch with the raw pixel data of both collected and generated (synthetic).

### 3.3. Loss Function and Evaluation Metrics

We used the binary cross-entropy [50] loss function as shown in Equation (1) to train the entire network:

(1) $$L_{c}=-\left(y\log(p)+(1-y)\log(1-p)\right)$$

where *y* is a binary indicator (0 or 1) if class label is the correct classification for the given instance and *p*—predicted probability that the given instance is of the class 1 (positive) or 0 (negative).

The expense of diagnosing a positive (abnormal) event as negative (normal) can threaten the system’s safety. Therefore, other performance measures must be used when the performance of the minority class is more critical than overall accuracy. The performance metrics such as recall, precision, area under the curve (AUC) score and F1-score are often used when the minority class is essential.

### 3.4. Evaluation Metric

With two labeled sets (actual or true and predictions), we can create a confusion matrix that will summarize the results of evaluation or inference phase of the classifier. The confusion matrix computed is a 2D matrix as shown in Table 1.

(2) $$Precision=\frac{TP}{TP+FP}$$

(3) $$Recall=\frac{TP}{TP+FN}$$

(4) $$Accuracy=\frac{TP+TN}{TP+FP+FN+TN}$$

(5) $$F1-score=2\times \frac{Precision\times Recall}{Precision+Recall}$$

The *F*1-score (in Equation (5)) is the harmonic mean of precision and recall with values between 0 and 1.

#### Receiver Operating Characteristics

A ROC curve is a graphical plot that depicts the diagnostic capability of a binary classifier system for different threshold values. Each point on the plot represents the ratio of the true positive rate (TPR) and the false positive rate (FPR) for various thresholds or cut-off points. Area Under the Curve (AUC) in the ROC curve is a threshold-independent metric that measures a binary classifier’s performance on classification problems. Normally, the classifier with a higher AUC is better than all other evaluated classifiers.

## 4. Experiment

We evaluated our approach on two different testing datasets and provided a meticulous comparison with three anomaly detection approaches.

### 4.1. Datasets Description

We obtained the datasets from a private company located in Seoul, South Korea. These datasets were named ‘Traindata1’, ‘Traindata2’, ‘Testdata1’, and ‘Testdata2’ consisting of 604,767, 604,767, 86,400, and 75,594 data points, respectively. The other dataset is synthetic, which was generated using aforementioned TimeSynth [49], a time-series synthetic data generator library.

### 4.2. Experiment Design

**Figure 2:**
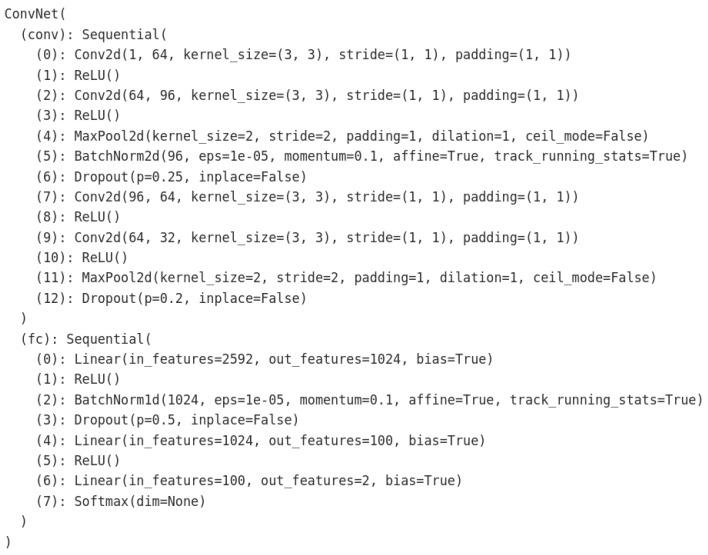
Our custom model architecture built as a Sequential Model which stacks series of feature extractors (2D-Convolutional Neural Networks), Activation function (ReLU), Down sampling operator (MaxPooling), BatchNormalization and Regularizer (Dropout), Classifier (Fully connected layer) that uses SoftMax function.

We designed a custom 2D-CNN as illustrated in Figure 2 and optimized it using Adam optimizer [27]. For the transfer learning, as we discussed, we adopted ResNet18 and then changed the last convolution layer and fully connected layer to match the required number of classes (that is two) for this case study. We compared our approach with Autoregressive Integrated Moving Average (ARIMA) [28], and Hierarchical Stacking Temporal Convolutional Network (HS-TCN) [29].

#### 4.2.1. ARIMA

Autoregressive Integrated Moving Average (ARIMA) models possess versatile properties in forecasting a time-series, leading to much adoption by research communities and industrial application developers. However, the ARIMA algorithm was designed for time-series forecasting, not classification problems. The ARIMA models are fitted to time-series data to understand the data reasonably or predict future points in the sequence (forecasting). Therefore, to make them compatible with the time-series anomaly detection problem, we check if the predicted value intervals lie outside the true value interval and then label it as an anomaly.

#### 4.2.2. HS-TCN

Hierarchical Stacking Temporal Convolutional Network (HS-TCN) is an ensemble learning method that integrates more than one classifier or regression model. HS-TCN is a method built on top of a powerful algorithm known as a Temporal Convolutional Network (TCN) [51] to solve sequential problems. It searches outliers, weeding them out as part of unlabeled data, and then labeling them as anomalies. As for the base classifiers, widely adopted base models are SVM, K-Nearest Neighbors (kNN), Bayesian networks and decision trees, which take the outliers out from the feature extracted by the TCN as input.

## 5. Results

For diverse comparison, we summarized the results computed as the accuracy (Acc), recall (RC), precision (PR), area under the curve (AUC) score and F1-score (F1) in several tables. We calculated the average of five runs to compute each performance measure metrics. We reported the average because the training instances were randomly selected from the entire training dataset.

### 5.1. Data Validity Analysis

Since the data are crucial for the model to learn informative features, they are worth careful and intensive exploration. This is because the required pseudo-class data should appropriately represent the abnormal target data (at least to our custom dataset). The model (Res1) showed poor performance in Table 2, which were the consequences of the model complexity (see Section 5.2) and the amount of training data.

We demonstrated the effect of the training data on learning which can be indicated from the experiment results of the ResNet models (Res3 and Res4 trained on normal and random noise) in Table 2, which underperformed the ResNet models (Res1) in Table 2.

As we can observe the results in Table 3, the custom model trained with only 2000 instances from each class outperformed the complex model (ResNet18) which experimentally proves that data played a big part in negatively or positively impacting the model performance.

From the several experiments conducted, it can see seen that:

- Using synthetic data as the pseudo-class is a promising direction because of the model performance improvement achieved after only changing the pseudo-class data from random or Gaussian noise to synthetic generated data.
- Transfer learning should be dealt with consideration that underfitting can happen because the transferred model is too complicated for training data (see Section 5.2).

Additionally, the experiments indicate that there exists a valid subspace where the representative samples of the target (synthetic) data are located, which makes it efficient for training the model. We improved the model performance from two aspects: (1) by using the data found for normal class, (2) and by efficiently and effectively exploiting that subspace for anomaly class.

We set up multiple configurations for the amount of data used for training as presented in Table 3. We obtained the highest scores in terms of F1-score and AUC when we configured the number of instances to be 2000 for each class.

### 5.2. Model Architecture Analysis

The appropriate model complexity (depth-wise) serves several important features (generalization, appropriate training, and inference time) of an efficient model. However, too complex models may either suffer from underfitting or overfitting depending on the training data. The ResNet’s performance results in Table 2 are not encouraging compared with custom model (in Figure 2) performance results in Table 3. As shown in the result tables, the complex model (such as ResNet) suffers from underfitting with the training data, leading to negative predictions for most data points in a test phase, hence the lowest Recall and F1-score. Thus, we investigated simple custom models that can capture enough information from the training data and generalize while mitigating the underfitting issue. As we can observe the results in Table 3, the custom model (in Figure 2) outperformed the complex model (ResNet18), which experimentally proves that the appropriate complexity of the model also greatly impacts the performance.

### 5.3. Comparison with Other Methods

In this subsection, we detail the performances of different approaches on our custom dataset. We summarized the results of several experiments in Table 4 computed using the F1-score and AUC score as performance metrics.

We further investigated the anomaly detection of the three selected approaches (ARIMA, HS-TCN, and our OC-CNN) by training them from scratch and analyzed their performances. We computed the mean (by repeatedly running each experiment five times using different seeds) for fair and correct evaluations, which is necessary and more suitable comparisons considering that most of the methods start with random weights. As we can see in Table 4, the 2D-Convolutional Neural Network-based anomaly detector OC-CNN (ours) significantly outperformed two approaches.

## 6. Discussion

### 6.1. Effectiveness of the OC-CNN

Performance metrics from the combination 2D-CNN custom model and synthetic data indicate that the CNN-based model can successfully achieve the objective of using pseudo-class and the simple model. Results indicate that the OC-CNN implemented by custom 2D-CNN outperforms the ResNet models and two other approaches. The reason for the high performance is that the feature representations learned were sufficiently relevant to distinguish between the two classes. This was possible because our 2D-CNN focused on understanding which patterns resemble normal or abnormal patterns instead of temporal features. At the same time, our 2D-CNN was able to learn sufficiently informative features from small training instances, which made it efficient yet effective for the presented case study. This effectiveness and efficiency allow companies to focus on other important core parts of the business and not to worry about the cost of data preparations. The data preparation requires a few cheap steps to generate, which do not require hours of training.

### 6.2. Limitation of the OC-CNN

Although the developed OC-CNN outperformed the other approaches in this case study, it has several drawbacks. It highly relies on the careful manual exploration of pseudo-class data generation. Therefore, improving and automating the process for synthetic data generation will be a more beneficial and highly reliable solution.

## 7. Conclusions

In this paper, we have investigated 2D Convolutional Neural Network technique and synthetic data for water-level anomaly detection. Our design for water-level anomaly detection intended to investigate various scenarios of training a simple model from scratch, transferring models across multiple domains and applying relevant synthetic (pseudo-class) data. We adopted those techniques based on their compatibility, simplicity, and capability to learn complex functions to represent an optimal subspace for normal and anomalous data. We experimentally demonstrated the effects of complexity of the models and amount/quality of the training data on model’s generalization performance. Additionally, we showed that a simple model with quality data is sufficient to learn from scratch a function that can discriminate the instances of the two classes in water-level anomaly detection. Unfortunately, it was not the same with a multi-domain transfer, whereby the ResNet model failed to perform effective classification and therefore underperform the simple custom model in this case study. However, despite the overall performance degradation of the ResNet, we showed that the relevant pseudo-class data could slightly improve the performance of the ResNet while leading to the higher performance of our OC-CNN model. This shows that time-series synthetic data could be one of the promising ways to solve the problem of lacking positive class data for anomaly detection. Despite the failure of multi-domain transfer, we still believe it is worth further investigation that would lead to an efficient model, which will be one of our future research directions.

## Figures and Tables

**Table 1:** The representation of the confusion matrix for binary classification task. True Positive (TP): the label and the prediction are positive, False Positive (FP): the label is negative, but the prediction is positive, False Negative (FN): the label is positive, but the prediction is negative, and True Negative (TN): the label and the prediction are negative.

	Predicted–Positive	Predicted–Negative
Actual–Positive	TP	FN
Actual–Negative	FP	TN

**Table 2:** Performance summary of ResNet models trained different on training data versions. Note: Number of training instances (ITs), ResNet models trained on custom and synthetic data, custom and Random noise data, custom and Gaussian noise data noted as Res1, Res2 and Res3, respectively, and the bold scores indicate the highest mean scores obtained in test phase.

	Dataset	ITs	Acc	PR	RC	F1	AUC
Res1	T1	8000	0.464	0.609	0.372	0.462	0.522
	T1	10,000	**0.830**	**0.828**	**0.392**	**0.494**	**0.564**
	T2	8000	0.893	0.793	0.569	0.662	0.595
	T2	10,000	**0.902**	**0.859**	**0.581**	**0.693**	**0.649**
Res2	T1	8000	0.884	0.706	0.186	0.294	0.509
Res3	T2	8000	0.818	0.232	0.235	0.234	0.505

**Table 3:** The results of the 2D custom model after trained on normal and synthetic data then tested with ‘Testdata1’ (noted as T1) and ‘Testdata2’ (noted as T2)’, the bold scores indicate the highest mean scores obtained in test phase. Note: Number of training instances (ITs).

Dataset	ITs	Acc	PR	RC	F1	AUC
T1	40,000	0.442	1.000	0.280	0.437	0.617
	10,000	0.901	1.000	0.664	0.798	0.798
	4000	88.902	1.000	0.641	0.781	0.623
	2000	**0.917**	**0.999**	**0.937**	**0.967**	**0.912**
T2	40,000	0.544	0.890	0.28	0.437	0.532
	10,000	0.839	0.901	0.693	0.798	0.624
	4000	0.968	0.983	0.845	0.887	0.841
	2000	**0.909**	**0.987**	**0.902**	**0.943**	**0.896**

**Table 4:** Performance comparison of different methods using F1-score (noted as F1) and AUC score averaged of five runs, the bold scores indicate the highest mean scores obtained in test phase, T1 (Testdata1) and T2 (Testdata2).

		ARIMA		HS-TCN		Our OC-CNN	
		F1	AUC	F1	AUC	F1	AUC
Dataset	T1	0.494	0.521	0.812	0.733	**0.963**	**0.922**
	T2	0.029	0.500	0.693	0.652	**0.954**	**0.887**

## Data Availability

The data that support the findings of this study are available from Infranics Co., Ltd., but restrictions apply to the availability of these data, which were used under license for the current study, and so are not publicly available. Data are however available from the authors upon reasonable request and with permission of Infranics Co., Ltd.

## Abbreviations

ARIMA	Autoregressive Integrated Moving Average Model
AUC	Area Under the Curve
CNN	Convolutional Neural Network
CPU	Central Processing Unit
DBN	Deep Belief Network
DNN	Deep Neural Network
AE	AutoEncoder
GAN	Generative Adversarial Network
IT	Information Technology
KNN	K-Nearest Neighbors
LSTM	Long Short-Term Memory
OCC	One-Class Classification
OC-SVM	One-Class Support Vector Machine
RNN	Recurrent Neural Network
SVVD	Support Vector Data Description
